# Supplementary material for: Effects of Bempedoic Acid in Acute Myocardial Infarction in Rats: No Cardioprotection and No Hidden Cardiotoxicity
Source: Int J Mol Sci. 2023 Jan 13;24(2):1585. doi: 10.3390/ijms24021585 (PMC9860765; doi:10.3390/ijms24021585)
Supplement: Supplementary file 1 [file ijms-24-01585-s001.zip › ijms-2113026-supplementary.pdf]

SUPPLEMENTARY MATERIAL

Article

Effects of bempedoic acid in acute myocardial infarction in rats: no cardioprotection and no hidden cardiotoxicity

Tamás G. Gergely# [1], Gábor B. Brenner# [1], Regina N. Nagy [1], Nabil V. Sayour [1], András Makkos [1], Csenger Kovácsházi [1], Huimin Tian [1], Rainer Schulz[2], Zoltán Giricz‡ [1,3], Anikó Görbe‡ * [1,3], Péter Ferdinandy‡ [1,3]

[1] MTA-SE System Pharmacology Research Group, Department of Pharmacology and Pharmacotherapy, Semmelweis University, H-1089 Budapest, Hungary
[2] Institute of Physiology, Justus Liebig University Giessen, 35390 Giessen, Germany
[3] Pharmahungary Group, H-6722 Szeged, Hungary

#,‡ These authors contributed equally.
* Correspondence: gorbe.aniko@med.semmelweis-univ.hu

Abstract: Lipid-lowering drugs have been shown to have cardioprotective effects but may have hidden cardiotoxic properties. Therefore, here we aimed to investigate if chronic treatment with the novel lipid-lowering drug bempedoic acid (BA) exerts hidden cardiotoxic and/or cardioprotective effects in a rat model of acute myocardial infarction (AMI). Wistar rats were orally treated with BA or its vehicle for 28 days, anesthetized and randomized to three different groups (vehicle + ischemia/reperfusion (I/R), BA + I/R, and positive control vehicle + ischemic preconditioning (IPC)) and subjected to cardiac 30 min ischemia and 120 min reperfusion. IPC was performed by 3 × 5 min I/R cycles before ischemia. Myocardial function, area at risk, infarct size and arrhythmias were analyzed. Chronic BA pretreatment did not influence cardiac function or infarct size as compared to the vehicle group, while the positive control IPC significantly reduced the infarct size. The incidence of reperfusion-induced arrhythmias was significantly reduced by BA and IPC. This is the first demonstration that BA treatment does not show cardioprotective effect although moderately reduces the incidence of reperfusion-induced arrhythmias. Furthermore, BA does not show hidden cardiotoxic effect in rats with AMI, showing its safety in the ischemic/reperfused heart.

Supplementary Figure 1.

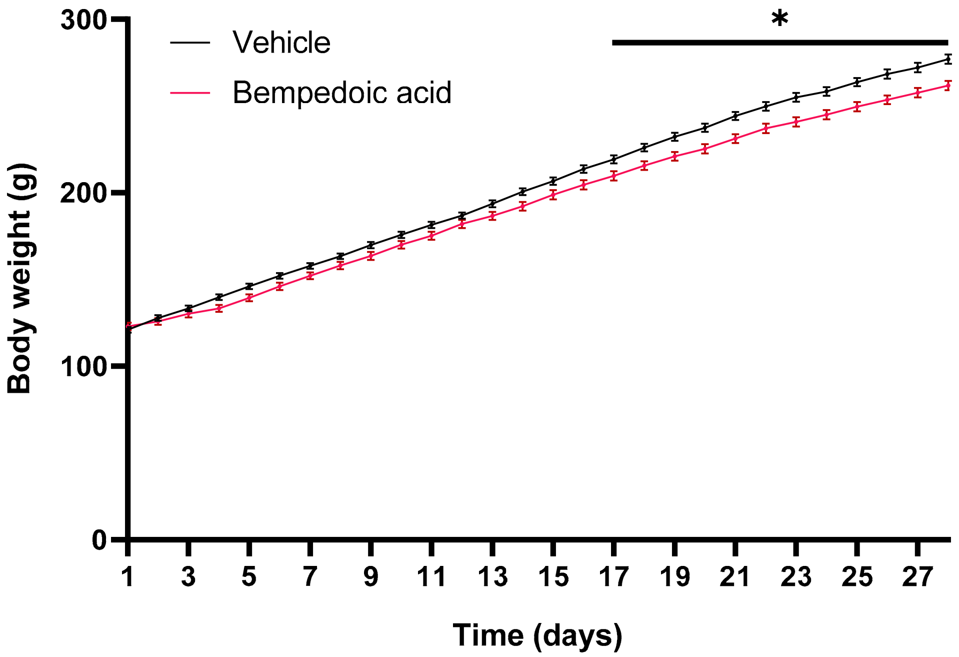

Body weights of animals during the 28 days treatment period. *$p < 0.05$ vs vehicle treated group, repeated measures ANOVA, Bonferroni's post hoc test, n = 26 for BA and n = 61 for vehicle treated groups.

Supplementary Figure 2.

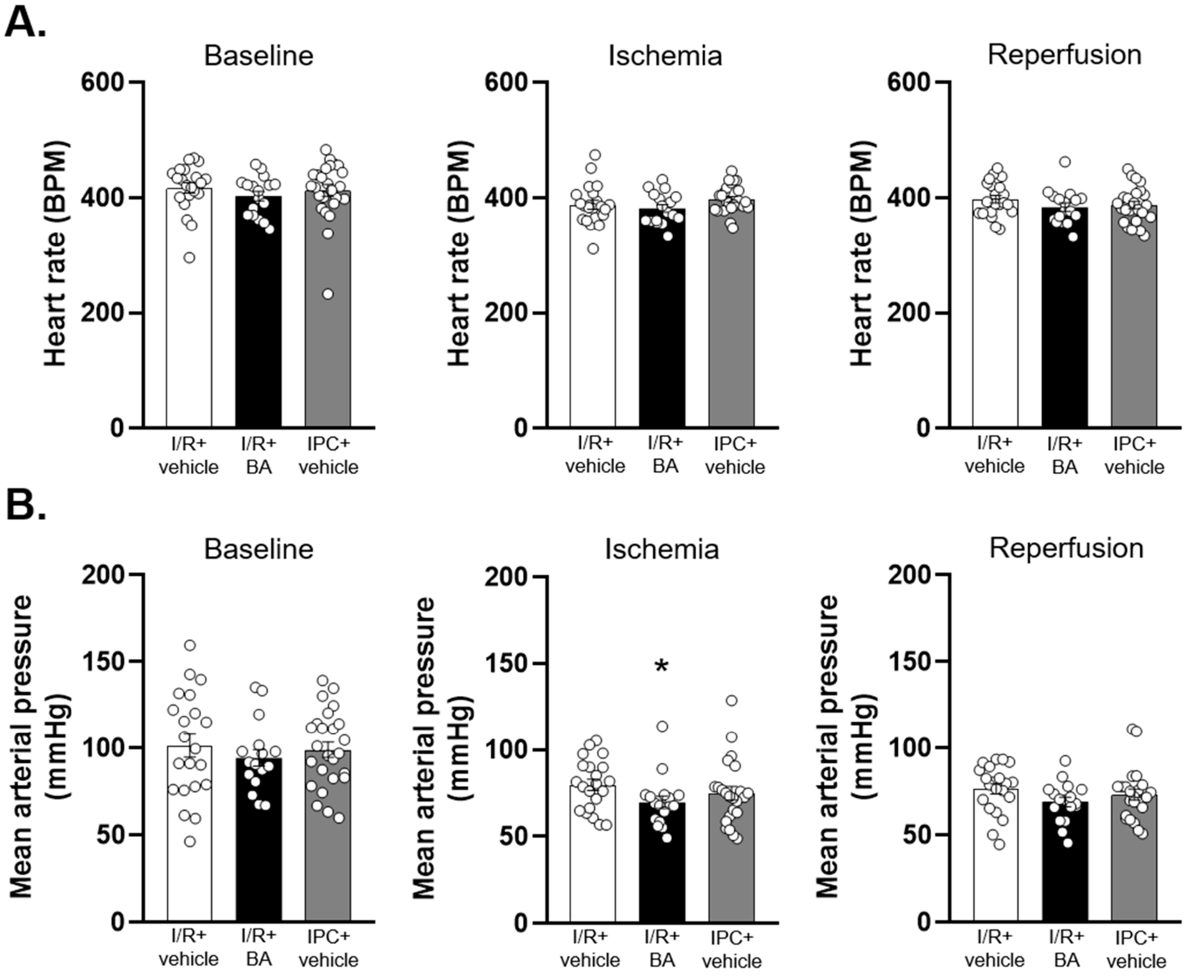

Heart rates (A) and mean arterial blood pressure (B) measured during surgery. Statistics: One-way ANOVA, followed by Tukey's post hoc test or Kruskal-Wallis test, followed by Dunn's post hoc test. *$p < 0.05$ vs I/R+vehicle group, n = 17-25 / group. BA: bempedoic acid IPC: ischemic preconditioning I/R: ischemia/reperfusion

Supplementary Table 1.

Parameter	Vehicle (n = 23)	Bempedoic acid (n = 18)
HR, 1/min	415.9 ± 6.1	424.6 ± 9.6
LVEDV, µl	283.0 ± 9.77	278.5 ± 7.79
LVESV, µl	99.4 ± 4.29	96.7 ± 5.06
LVIDd, mm	6.32 ± 0.10	6.32 ± 0.08
LVIDs, mm	3.41 ± 0.10	3.31 ± 0.09
LVAWd, mm	1.91 ± 0.06	1.86 ± 0.04
LVAWs, mm	3.06 ± 0.04	3.01 ± 0.08
LVPWd, mm	1.76 ± 0.05	1.76 ± 0.04
LVPWs, mm	2.74 ± 0.06	2.81 ± 0.04
LV mass, mg	627.7 ± 15.7	613.4 ± 12.5
SV, µl	183.5 ± 7.2	181.8 ± 6.4
EF, %	64.8 ± 0.96	65.3 ± 6.38
FS, %	46.2 ± 0.92	47.7 ± 1.09
CO, ml/min	76.1 ± 2.97	76.7 ± 2.54
E, cm/s	830.8 ± 22.9	826.3 ± 24.0
e', cm/s	51.6 ± 3.12	59.9 ± 3.54
E/e'	17.0 ± 1.11	14.6 ± 0.95
IVCT, ms	14. 4 ± 0.42	14.3 ± 0.70
IVRT, ms	24.8 ± 0.50	24.6 ± 0.56

Measured and calculated echocardiographic parameters after 28 days of vehicle or bempedoic acid treatment. All values are represented as means ± SEM. Statistics: Student's *t* test, n.s. HR, heart rate; LVEDV, left ventricular (LV) end-diastolic volume; LVESV, LV end-systolic volume, LVIDd and LVIDs, LV internal diameters at diastole and systole, respectively; LVAWd and LVAWs, LV anterior wall thickness at diastole and systole, respectively; LVPWd and LVPWs, LV posterior wall thickness at diastole and systole, respectively; SV, stroke volume; EF, ejection

fraction; FS, fractional shortening; CO, cardiac output; E, peak Doppler blood inflow velocity across the mitral valve during early diastole; e', peak tissue Doppler of myocardial relaxation velocity at the mitral valve annulus during early diastole; IVCT, isovolumic contraction time; IVTR, Isovolumic relaxation time.

Supplementary Table 2.

	**Arrhythmia type**	**Vehicle + I/R (n=26)**	**BA + I/R (n=24)**	**Vehicle + IPC (n=27)**
**Ischemia**	VPB incidence	199.3 ± 30.1	180.3 ± 28.7	20.1 ± 10.5 * #
	VT incidence	8.3 ± 2.2	7.6 ± 1.3	0.03 ± 0.03 * #
	VT duration, s	35.9 ± 11.0	25.8 ± 5.4	0.05 ± 0.05 * #
	NSVT incidence	7.9 ± 2.1	7.3 ± 1.3	0.04 ± 0.04 * #
	NSVT duration, s	17.5 ± 5.4	15.7 ± 3.2	0.05 ± 0.05 * #
	SVT incidence	0.5 ± 0.1	0.3 ± 0.1	0 ± 0 * #
	SVT duration, s	18.3 ± 7.2	10.2 ± 4.2	0 ± 0 * #
	VF incidence	0.4 ± 0.2	0.4 ± 0.1	0 ± 0 #
	VF duration, s	2.3 ±1.6	10.4 ± 6.3	0 ± 0 #
**Reperfusion**	VPB incidence	8.5 ± 1.5	8.8 ± 1.3	5.8 ± 1.5
	VT incidence	1.1 ± 0.3	0.4 ± 0.2*	0.1 ± 0.06 *
	VT duration, s	6.0 ± 1.7	3.1 ± 1.9	0.2 ± 0.1 *
	NSVT incidence	0.9 ± 0.2	0.3 ± 0.2 *	0.1 ± 0.06 *
	NSVT duration, s	3.2 ± 1.0	2.5 ± 1.7	0.2 ± 0.1*
	SVT incidence	0.2 ± 0.1	0.1 ± 0.1	0 ± 0
	SVT duration, s	2.7 ± 1.1	0.6 ± 0.6	0 ± 0 *
	VF incidence	0 ± 0	0 ± 0	0 ± 0
	VF duration, s	0 ± 0	0 ± 0	0 ± 0

Incidence and duration of arrhythmias during ischemia and the first 15 minutes of reperfusion. All values are represented as means ± SEM. Statistics: Kruskal-Wallis test, followed by Dunn's post hoc test. *$p < 0.05$ vs I/R+vehicle group, #$p < 0.05$ vs I/R+BA group..

BA: bempedoic acid IPC: ischemic preconditioning I/R: ischemia/reperfusion
